# A Ru/RuO_2_-Doped TiO_2_ Nanotubes as pH Sensors for Biomedical Applications: The Effect of the Amount and Oxidation of Deposited Ru on the Electrochemical Response

**DOI:** 10.3390/ma14247912

**Published:** 2021-12-20

**Authors:** Nikola Macháčková, Jitřenka Jírů, Vojtěch Hybášek, Jaroslav Fojt

**Affiliations:** Department of Metals and Corrosion Engineering, Faculty of Chemical Technology, University of Chemistry and Technology in Prague, Technická 5, 166 28 Prague, Czech Republic; machacki@vscht.cz (N.M.); jiruj@vscht.cz (J.J.); hybasekv@vscht.cz (V.H.)

**Keywords:** pH sensor, TiO_2_ nanotubes, ruthenium, Mott–Schottky, electrochemical impedance spectroscopy

## Abstract

In the field of orthopedic or dental implants, titanium and its alloys are most commonly used because of their excellent mechanical and corrosion properties and good biocompatibility. After implantation into the patient’s body, there is a high risk of developing bacterial inflammation, which negatively affects the surrounding tissues and the implant itself. Early detection of inflammation could be done with a pH sensor. In this work, pH-sensitive systems based on TiO_2_-Ru and TiO_2_-RuO_2_ combinations were fabricated and investigated. As a base material, Ti-6Al-4V alloy nanostructured by anodic oxidation was used. Ruthenium was successfully deposited on nanotubular TiO_2_ using cyclic polarization, galvanostatic and potentiostatic mode. Potentiostatic mode proved to be the most suitable. The selected samples were oxidized by cyclic polarization to form a TiO_2_-RuO_2_ system. The success of the oxidation was confirmed by XPS analysis. The electrochemical response of the systems to pH change was measured in saline solution using different techniques. The measurement of open circuit potential showed that unoxidized samples (TiO_2_-Ru) exhibited sub-Nernstian behavior (39.2 and 35.8 mV/pH). The oxidized sample (TiO_2_-RuO_2_) containing the highest amount of Ru exhibited super-Nernstian behavior (67.3 mV/pH). The Mott–Schottky analysis proved to be the best method. The use of the electrochemical impedance method can also be considered, provided that greater stability of the samples is achieved.

## 1. Introduction

The introduction of an implant into the human body can be accompanied by a number of risks and complications. One of them is the development of inflammation at the implant–tissue interface, which can cause chronic diseases, damage to the surrounding tissue or implant failure by corrosion. In many cases, the inflammation is detected too late and the patient has to undergo reoperation, which is very burdensome [1,2,3,4,5]. The cause of the inflammation is the accumulation of various types of bacteria forming a biofilm, whose subsequent metabolic activity leads to a decrease in the pH around the inflammation. The formation of bacterial biofilm is initiated by the adhesion of bacteria on the implant surface followed by their accumulation in multiple layers. The decisive parameter at this stage is the wettability of the surface. Tang et al. [6] prepared TiO_2_ nanotubes with superhydrophobic properties, in which *S*. *aureus* colonization occurred only in minimal amounts. The biofilm then matures, this process is accompanied by the formation of substances such as teichoic acid, exopolysaccharides and others. In the last stage, the bacteria switch back to a planktonic state in which they colonize the new surface [2,6,7,8]. *T. denticola*, *T. forsythia*, *S. mutans* or *C*. *albicans* whose acidic metabolic products are the cause of periimplantitis are mainly found in the area of dental implants [5,9,10]. The most common bacteria in the orthopedic implant area are *S*. *mutans*, *S*. *epidermidis* and aerobic *E*. *coli*. In particular, facultative anaerobic staphylococci predominate, because they are able to switch to fermentative metabolism in the absence of oxygen, leading to the formation of lactic acid, acetic acid or formate [2,11,12]. Current research is focused on the development of pH sensors that would be able to detect the occurrence of inflammation in the initial stage [13].

Ion-sensitive metal oxides (MO_x_) are the focus of research interests in the development of pH sensors. These oxides with micro/nanostructured morphology exhibit an exceptional combination of properties with a large ratio between real surface area and sample size [14,15]. There are various methods for the preparation of MO_x_ pH sensors, which include sputtering [16,17], electrodeposition [18,19], anodic oxidation [20,21], screen printing [22,23] and others. According to the method of sensing the electrochemical processes, pH sensors are divided into potentiometric, chemoresistive, ISFET (ion-sensitive field-effect transistor), EGFET (extended-gate field effect transistor) and conductometric/inductive/capacitive sensors [14]. Following oxides and their possible combinations are commonly used: TiO_2_, RuO_2_, PdO, IrO_2_, MnO_2_, Ta_2_O_5_, ZnO and WO_3_.

Zhao et al. [20] prepared a pH sensor based on amorphous or anatase TiO_2_ nanotubes. Amorphous nanotubes showed a better response to pH change, close to Nernst behavior (59.3 mV/pH), after irradiation their wettability increased. Similar results were obtained by Monteiro et al. [21]. Manjakkal et al. [22] prepared a potentiometric pH sensor with a RuO_2_-TiO_2_ based sensing electrode. The potential gradually decreased with increasing pH; this dependence was linear with the slope 56.1 mV/pH.

In their other work, Manjakkal et al. [24] prepared a pH sensor based on a thick film of RuO_2_ which they investigated using electrochemical impedance spectroscopy (EIS). This analysis revealed that the response of the system is strongly dependent on the concentration of H^+^ and OH^−^ ions adsorbed on the surface. The relationship between the pH of the solution and sensor oxide parameters (*R*_s_, *R*_ct_, and *C*_dl_) was investigated. In the low frequency region, the greatest dependence of capacity, conductivity and impedance on pH was demonstrated. The research by Simić et al. [25] focused on a pH sensor consisting of a thick film of TiO_2_. Analysis of this sensor using EIS showed similar results as previous work. Charge transfer and ion exchange emerged as dominant phenomena in sensing pH changes.

Hara and Sugimoto [26] used Mott–Schottky analysis (MS) to study a semiconductor pH sensor consisting of TiO_2_ doped with Nb_2_O_5_. The measured curves were linear at all pH, the value of the slope did not change significantly, indicating that it is independent on pH. The flat-band potential as a function of pH was calculated using Formula (1).

*E*_fb_ = *ϕ* − 4.5 + (2.303*RT*/*F*) × (pH_pzc_ − pH), (1)

*E*_fb_—flat-band potential, *ϕ*—work function of the semiconductor, −4.5—the energy difference between the vacuum and the hydrogen reference electrode, pH_pzc_—pH at zero charge. The calculated flat-band potential decreased with increasing pH.

The present work aimed to prepare pH sensors based on TiO_2_ nanotubes with deposited particles of ruthenium under different conditions and then to determine the effect of pH change on the electrochemical response of the prepared surfaces. Oxidation was performed on selected samples to prepare a surface system with the combined effect of two oxides (TiO_2_-RuO_2_). Electrochemical response to pH change was monitored by various methods (measuring open circuit potential, EIS and MS).

## 2. Materials and Methods

Samples of Ti-6Al-4V ELI (extra low interstitials) alloy of cylindrical character with a diameter of 15 mm and a thickness of 3 mm were used as the base material. Specimens were water ground up to FEPA P2500 paper, then rinsed with distilled water, ethanol and acetone. Finally, they were dried with a stream of hot air. TiO_2_ nanotubes on the sample’s surface were prepared by anodic oxidation at room temperature in the electrolyte which contained 1 mol/L (NH_4_)_2_SO_4_ and 0.2 wt.% NH_4_F. A Jaissle Potentiostat-Galvanostat IMP 88 PC-200V with controlling unit PGU-AUTO Extern was used. Parameters of anodic oxidation were set by EcmWin software. The experiment was carried out in PTFE cell using a standard three-electrode setup: Ag/AgCl reference electrode (3 mol/L KCl, ACLE), glassy carbon counter electrode and sample as a working electrode. In the first phase of anodic oxidation, the potential was increased to 20 V/ACLE at a rate of 100 mV/s. This was followed by the potentiostatic phase of 2000 s. After that, samples were cleaned with distilled water and ethanol in an ultrasonic bath and dried with acetone and hot air.

Ruthenium particles were deposited on TiO_2_ nanotubes from 0.002 mol/L RuCl_3_ solution with pH = 1. In this work, three different principles of deposition were tested:

- galvanostatic mode (GS, *I* = −50 mA/cm^2^, *t* = 1200 s);
- cyclic polarization (CP, potential range 1 V/ACLE to −1 V/ACLE, rate: 10 mV/s);
- potentiostatic mode (PS, *E* = −0.7 V/ACLE, *t* = 600 or 1200 s).

The exposed area of 1 cm^2^ was set by o-ring. Depositions were performed with the same setup as anodic oxidation in an electrochemical cell with a 30 mL volume of solution. Part of the prepared samples was oxidized to obtain a stable RuO_2_. Firstly, the CP in 1 mol/L H_2_SO_4_ in the potential range 0.07 V/ACLE–0.97 V/ACLE with polarization rate 1 mV/s was performed. Then samples were annealed at 150 °C for 2 h. To determine the total amount of deposited Ru, the blind measurement in NaCl solution with the same pH and containing the same concentration of Cl^−^ as used RuCl_3_ solution was carried out.

For subsequent electrochemical testing, samples prepared in the potentiostatic mode were selected and will be labeled as follows:

- PS 600 (*E* = −0.7 V/ACLE, *t* = 600 s);
- PS 600ox (*E* = −0.7 V/ACLE, *t* = 600 s + oxidation by cyclic polarization, annealing);
- PS 1200 (*E* = −0.7 V/ACLE, *t* = 1200 s);
- PS 1200ox (*E* = −0.7 V/ACLE, *t* = 1200 s + oxidation by cyclic polarization, annealing).

Scanning electron microscope (SEM) TESCAN VEGA 3 LMU with OXFORD INCA 350 EDS analyzer was used for morphological analysis of prepared samples. The diameter of the nanotubes was measured via SEM images in ImageJ software. The surface characterization was carried out by X-ray photoelectron spectroscopy (XPS) analysis. ESCAprobe P (Omicron Nanotechnology Ltd.) with Al (K_α_ = 1486.7 eV) radiation source was used. The spectra were measured with an energy step of 0.05 eV. Individual binding energies were calibrated to a C 1s peak (285 eV). Results were processed using CasaXPS software, NIST X-ray Photoelectron Spectroscopy Database [27] and X-ray Photoelectron Spectroscopy References Pages [28].

All electrochemical measurements in this work were realized with potentiostat Gamry Instrument Reference 600 at temperature 37 °C. Samples were placed into an electrochemical cell with the same setup as was mentioned earlier. The exposed area was 1 cm^2^. The results were processed in Gamry Echem Analyst. As the exposure medium, saline solution (9 g/L NaCl) with equilibrium oxygen concentration buffered with biological buffer TES (N-Tris(hydroxymethyl)methyl-2-aminoethanesulfonic acid) in concentration 5.9 g/L was used. The pH of the solution was gradually reduced from 7.8 to 5.5 with diluted HCl, the reproducibility of the results was verified by a subsequent increase back to 7.8 with NaOH solution. The electrochemical response of the reference sample (nanostructured Ti-6Al-4V) and prepared systems with deposited Ru on changes of pH was studied. Open circuit potential (*E*_OC_), impedance response using the EIS method and MS curves were studied. Electrochemical impedance spectroscopy is a measuring method using AC voltage excitation of the sample. Based on the impedance response, the phase boundary could be simulated by an equivalent electrical circuit (EC) and allow for a more detailed description of the sample–electrolyte system. The Mott–Schottky analysis is also an AC technique, which measures the dependence of the reciprocal square capacitance on the selected frequency at different polarizations. This allows the semiconductor type and number of charge carrier evaluations. The scheme and conditions of the whole measurement are shown in Table 1. The calculation of the mean value and confidence interval (95%) was used to process the measurements results.

## 3. Results and Discussion

### 3.1. Preparation of TiO_2_ Nanotubes

**Figure 1:**
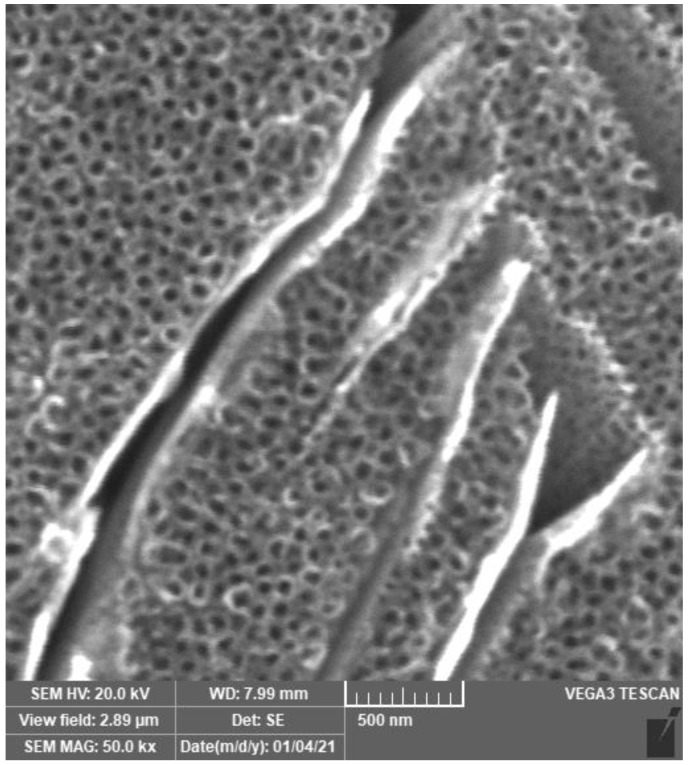
The nanostructured surface of Ti-6Al-4V prepared by anodic oxidation.

The anodic oxidation method for the formation of nanotubular TiO_2_ on titanium alloys is generally known and has been used in many studies [29,30,31,32]. The morphology of prepared nanotubes is strongly dependent on applied potential, time of exposition and composition of the electrolyte. TiO_2_ nanotubes prepared in this work are shown in Figure 1. It is evident that the nanostructure on Ti-6Al-4V is not homogenous, etched areas of β-phase (body-centered cubic structure) are present. This is because the β-phase is enriched with vanadium, whereas the α-phase (hexagonal close-packed structure) is enriched with aluminum. In a fluoride environment, vanadium dissolves faster than aluminum and for this reason, the formation of nanotubes in the surface is not uniform. Nanotubes in the area of β-phase are shorter and located deeper [33]. The inner diameter of nanotubes ranged from 20 to 80 nm with the most frequent diameter range of 55–60 nm. These results are close to those obtained in the work of Filova et al. [34].

### 3.2. Comparison of Deposition Methods of Ruthenium

**Figure 2:**
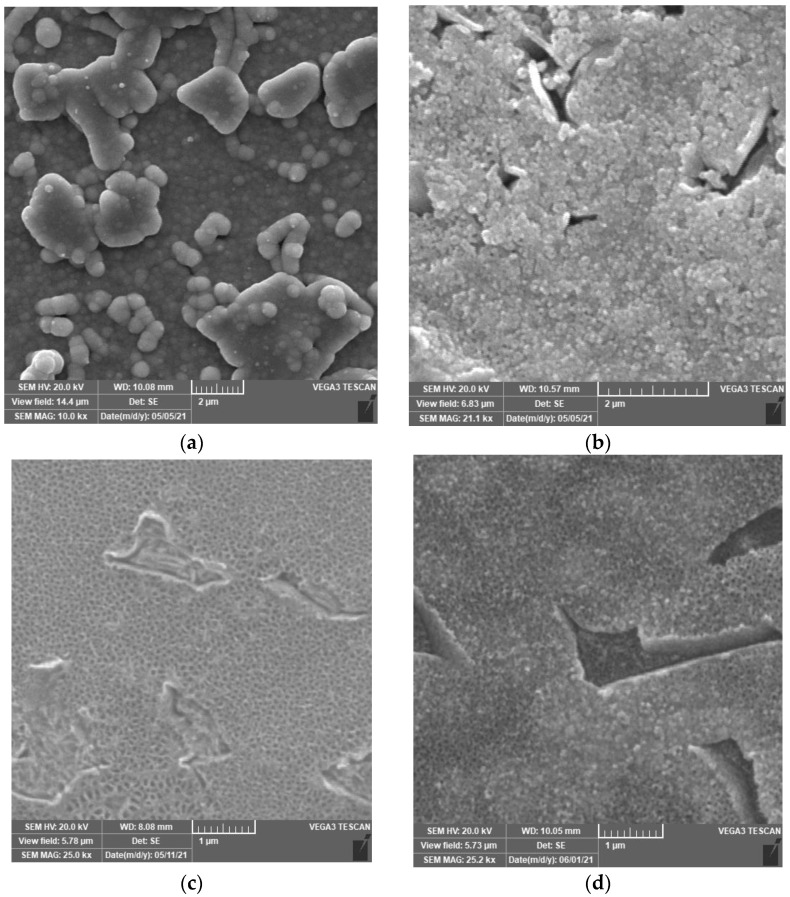
TiO_2_ nanotubes with deposited particles of Ru by different methods: (**a**) galvanostatic mode; (**b**) cyclic polarization; (**c**) potentiostatic mode (t = 600 s); (**d**) potentiostatic mode (t = 1200 s).

Different deposition methods of ruthenium particles were tested to obtain their optimum combination with TiO_2_ nanotube substrate. The principle of ruthenium deposition has been studied in many scientific papers [35,36,37]. Ru^3+^ ions are reduced on the substrate surface to metallic Ru. Due to this reaction, gas hydrogen evolves on the cathode. This process leads to a local increase in pH. The evolution of hydrogen bubbles is crucial for the cathodic deposition of many metals (Cu, Ru, Zn and Ag). Hydrogen bubbling disrupts the diffusion layer and increases the limit diffusion current density, which has a profound effect on growth morphology. The dendritic structure of deposited Ru was prepared by Oppedisano et al. [35] because of the application of high potential values (−4 up to −5 V/ACLE). The local increase of pH due to the consumption of H^+^ ions for H_2_ evolution initiates the formation of Ru(OH)_3_. The hydroxide is undesirable because it is not very stable compared with RuO_2_. For this reason, a solution of pH = 1 was used to avoid significantly exceeding this limit. To convert Ru to RuO_2_, cyclic polarization in H_2_SO_4_ (1 mol/L) was performed in the potential range 0.07 V/ACLE–0.97 V/ACLE. Nanostructured samples of Ti-6Al-4V with deposited Ru prepared by cyclic polarization (CP), galvanostatic (GS) and potentiostatic (PS) mode are shown in Figure 2.

**Figure 3:**
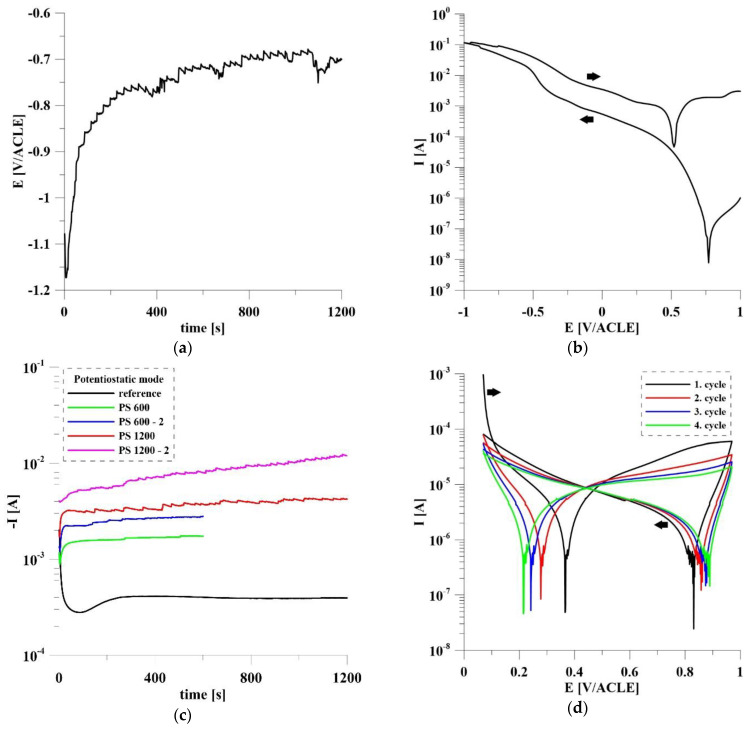
The current and potential dependencies of individual modes of Ru deposition: (**a**) galvanostatic mode (**b**) cyclic polarization; (**c**) potentiostatic mode; (**d**) oxidation of sample PS 1200ox by cyclic polarization in H_2_SO_4_.

The current and potential dependencies of the individual Ru deposition modes and oxidation by cyclic polarization of sample PS 1200ox are shown in Figure 3. The biggest amount of Ru was deposited using galvanostatic mode (−50 mA/cm^2^, 1200 s). This was confirmed by EDS analysis of the samples, the results of which are shown in Table 2. Figure 3a shows that galvanostatic deposition took place all the time in the potential range −1.2 to −0.7 V/ACLE. This means that at pH = 1 the potential was below the water stability zone at all times and thus H_2_ evolves intensively. This fact and the high applied current led to the formation of thick Ru film (Figure 2a). Cyclic polarization (from 1 V/ACLE to −1 V/ACLE, 10 mV/s) also led to a large amount of deposited Ru, which covered TiO_2_ nanotubes and was found out in etched β-phase. From the cyclic polarization course (Figure 3b) it is clear that, after reaching potential near −0.4 V/ACLE, there was an order of magnitude increase in current (maximum *I* = 123 mA). The anodic curve is shifted towards higher current values, indicating that the anodic oxidation of Ru^3+^ ions contributes significantly to the growth of Ru film. Jow et al. [37] confirmed that this method leads directly to the deposition of RuO_2_. In Figure 3c courses of potentiostatic deposition and reference blind measurement are shown. The total charge of the blind experiment was *Q* = −476.5 mC. The total amount of the deposited Ru was calculated by Faraday’s law from total charge minus charge from blind measurement (only hydrogen reduction). The calculated amount of electrochemically deposited Ru by the PS method is summarized in Table 3. Due to the complex nanostructured surface, uniform results in Ru deposition were not achieved. The emerging hydrogen gas may also have a large influence, the effect of which is not uniform and cannot be controlled much due to the location of the particles that are also inside the nanotubes. PS deposition gave the optimal amount of Ru particles. Our goal was to combine the effect of Ru/RuO_2_ with TiO_2_ nanotubes. Other methods (GS, CP) gave a thick, nonuniform film that covered the nanotubes. A smaller amount of Ru and a thinner layer is better for applications in the human body because less particles of Ru could be extracted from the implant into the tissues. The results of the EDS analysis show that fluorine was present in almost all samples. This is caused by anodic oxidation of Ti-6Al-4V in a solution containing NH_4_F to prepare nanotubes [34,38]. Figure 3d shows the example of the course of oxidation by cyclic polarization in 1 mol/L H_2_SO_4_ in the potential range 0.07 V/ACLE–0.97 V/ACLE.

### 3.3. Surface Characterization by XPS Analysis

The chemical composition of the surface of PS 1200 and PS 1200ox determined by XPS analysis is summarized in Table 4. The carbon and oxygen from the atmosphere contamination were not included in the analysis. Because of the overlapping peaks of the individual elements (Ti 2p and Ru 3p, Ru 3d and C 1s), their representation in at.% was calculated using the Formula (2) [39]:

(2) $$c_{x}=\frac{I_{x}/S_{x}}{\sum^{\;}I_{i}/S_{i}},$$

*c_x_*—concentration of the element, *I*—peak area, *S*—relative sensitivity factor. Then the wt.% was calculated.

**Figure 4:**
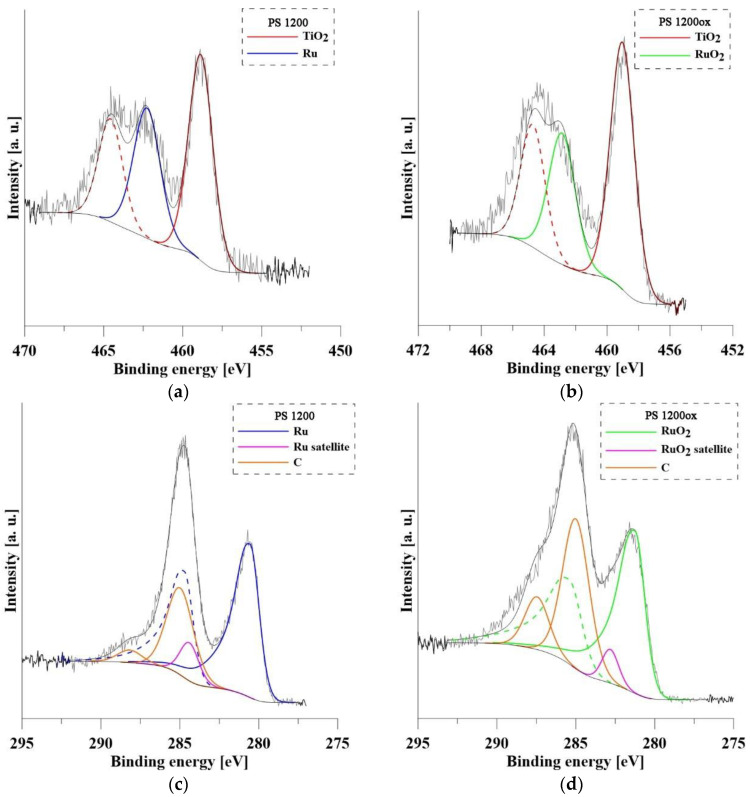
XPS spectra of Ti and Ru (PS 1200 and PS 1200ox): (**a**) Ru 3d and C 1s peaks—PS1200; (**b**) Ru 3d and C 1s peaks—PS1200ox; (**c**) Ru 3p and Ti 2p peaks—PS1200; (**d**) Ru 3p and Ti 2p peaks—PS1200.

The Al content is higher compared with the declared composition of the Ti-6Al-4V alloy, whereas V was not detected in the surface layer. This phenomenon is due to the higher affinity of Al for oxygen compared to Ti. Vanadium has a significantly lower affinity for oxygen than Ti [40]. Al was present in the form of Al_2_O_3_ (Al 2p peak: 74.5 eV). The Ru content in the surface layer is higher in PS 1200, which is not consistent with the results of the EDS analysis. This indicates that the Ru particles are largely deposited deeper in the nanotube cavity where they were detected by EDS analysis. Another reason is that oxidation by cyclic polarization in H_2_SO_4_ (1 mol/L) caused the partial dissolution of Ru particles located on the surface. The record of oxidation of PS 1200ox is shown in Figure 3d. It is clear that two types of chemical reactions were taking place during this process. In the range of potentials 0.2–0.4 V/ACLE, Ru was dissolved to the form of Ru^3+^, while in the range of 0.8–0.9 V/ACLE Ru was oxidized to RuO_2_. The work of Sugawara et al. [41] shows that the most intense dissolution of Ru occurs when the potential exceeds 0.7 V/ACLE, when RuO_2_ emerges from RuO that is formed on lower potentials. After the formation of stable RuO_2_, further dissolution is blocked. The spectra of Ti and Ru are shown in Figure 4. The doublets to the respective peaks are indicated by dashed lines. Ti was, as expected, in both samples present as TiO_2_. In its spectra, there was an overlap with Ru 3p peak (462.2 eV) and RuO_2_ (462.8 eV) on the oxidized sample PS 1200ox. On the not oxidized sample PS 1200 Ru 3d was present only in form of metallic Ru (280.5 eV). For PS 1200ox there is a clear shift in the spectra, with Ru 3d present as RuO_2_ (281.3 eV). For both spectra, there was an overlap with C 1s peaks from the contamination.

### 3.4. Electrochemical Response to pH Change

**Figure 5:**
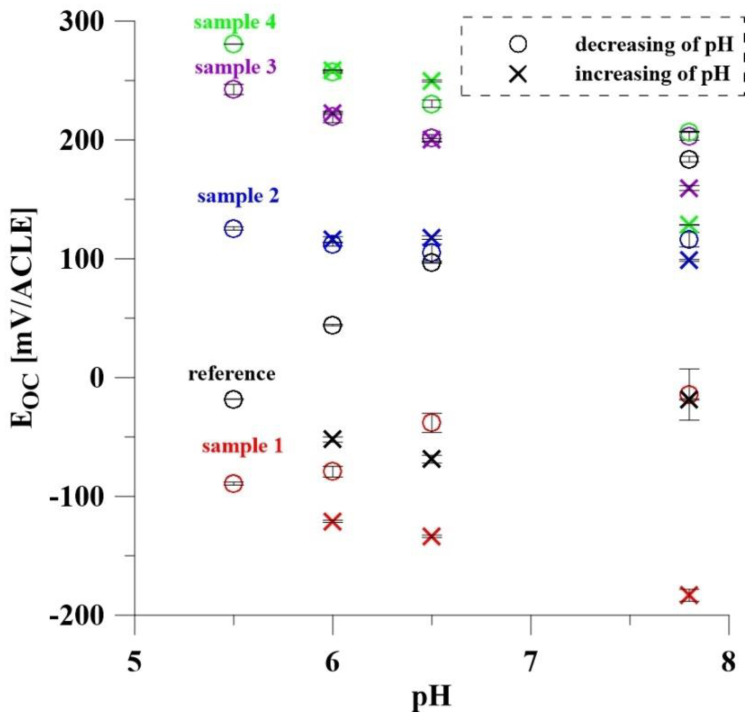
Calculated average E_OC_ values of all samples as a function of pH.

The electrochemical response of clear TiO_2_ nanotubes and those with deposited Ru (the PS samples) on pH change was measured. The pH values of 7.8 and 6.5 were chosen because inflammation in the human body can cause the ambient pH to drop by approximately one pH unit [42]. The subsequent values of 6.0 and 5.5 are already quite extreme and are not reached in the area of inflammation. These values were measured to obtain more values and to determine the behavior of the individual surfaces. The aim was to compare the effect of the amount of deposited Ru and its oxidation on the sensitivity of the system. Measurement of the open circuit potential for individual samples showed inconsistent results (Figure 5). For the reference sample, the decrease in pH led to a decrease in E_OC_. This dependence does not correspond to the typical Nernstian behavior of oxides. The probable reason for this is the presence of fluorides in the structure of TiO_2_ nanotubes, which are the residue after anodic oxidation. The unoxidized samples (PS 600, 1200) showed a sub-Nernstian behavior (39.2 and 35.8 mV/pH). Based on the results of the XPS analysis, which showed that Ru is present only in its metallic form on these samples, it can be concluded that the TiO_2_ nanostructure has a greater influence on the E_OC_. For the oxidized samples a different behavior was observed depending on the amount of RuO_2_ on the surface. Sample PS 600ox, with significantly less RuO_2_ content, did not show a linear dependence of E_OC_ on pH, for this reason, it was not possible to determine the slope dependence. In contrast, PS 1200ox showed a linear behavior both with decreasing (31.2 mV/pH) and increasing (67.3 mV/pH) pH. The inconsistency of the results for this sample can be explained by some variability of the samples over time. This may be due to a large amount of Cl^−^ ions in solution at the lowest pH, which reacted with Ru to form complex compounds whose presence affected subsequent results during the pH increase from 5.5 to 7.8. The pH-sensitive RuO_2_-TiO_2_ thick film electrodes prepared by Manjakkal et al. [22] achieved better sensitivity (56.11 mV/pH) compared with most of the samples prepared in this work, which is expected given the more uniform surface. We aimed to prepare a thin layer of Ru to limit its amount in the patient’s body; moreover, thin films are more durable compared to thick ones.

**Figure 6:**
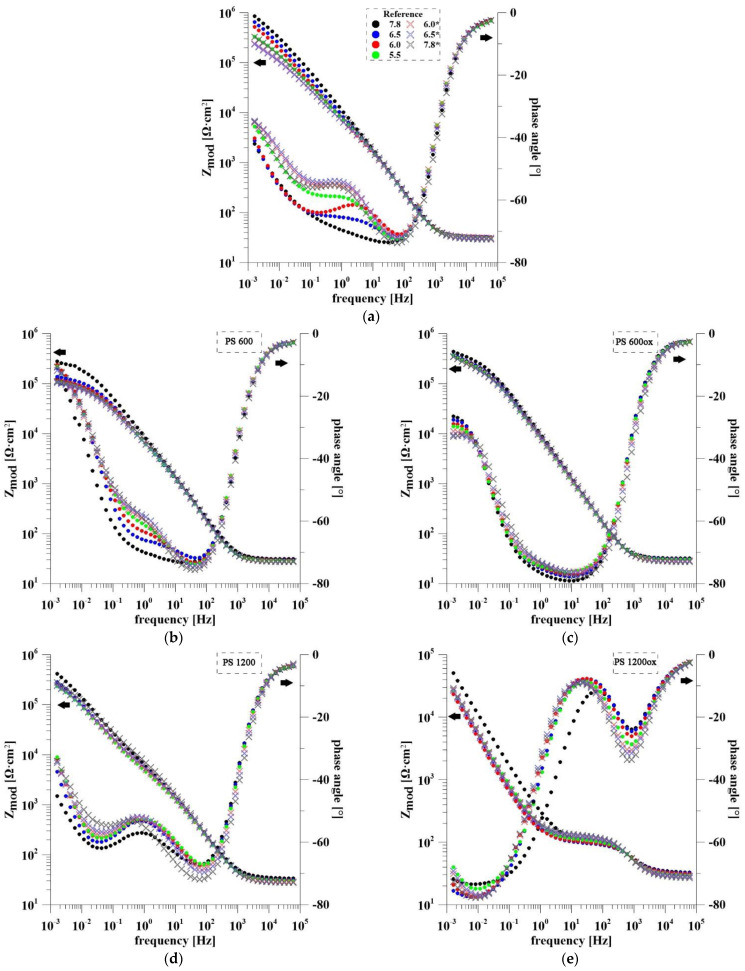
EIS spectra of all samples in physiological solution during pH changing: (**a**) Reference sample; (**b**) PS 600; (**c**) PS 600ox; (**d**) PS1200; (**e**) PS 1200ox.

A typical method for detecting pH change is the measurement of *E_OC_* mentioned above. This work aimed to try other possible electrochemical techniques such as EIS and also MS. Electrochemical impedance spectroscopy also allows a rather detailed analysis of the behavior of porous electrodes and nanomaterials. The Bode impedance plots of all samples are shown in Figure 6. The curves measured during the pH increase from 5.5 to 7.8 are marked with an asterisk in the legend. It is clear from the plots for the reference sample that the TiO_2_ nanotubes showed a time evolution independent of the pH change. The spectrum measured first at pH = 7.8 has a different shape compared with the other measurements. The typical shape of the nanostructure is not fully apparent here. This phenomenon is due to the slow flooding of the nanotubes. The decrease in impedance modulus in the low frequency region indicates decreasing corrosion resistance of the sample. The significant effect of TiO_2_ nanostructure on the electrochemical response of samples PS 600 and PS 1200 is evident from their spectra, especially in the mid-frequency region. For both spectra, the dependence of the phase angle on the pH at frequency 1 Hz is evident, which was achieved in the first half of exposure. From pH 7.8 to 5.5, the phase angle varied in the range −70° to −61° for PS 600 and −57° to −52° for PS 1200. Subsequently, increasing pH did not confirm this trend. It is obvious that samples with metallic Ru on the surface are not stable, changing over time, and therefore they are not useful for the detection of pH changes by EIS under these conditions. From the impedance response of PS 600ox and PS 1200ox it is clear that their behavior is quite different, implying that the amount of RuO_2_ is crucial. The dependence of the phase shift on pH is evident in the low and mid frequency region for PS 600ox. At a frequency of 10 Hz, the phase shift increases from −79° to −76°. For PS 1200ox, the spectrum measured for the first time at pH = 7.8 behaves differently compared with the others, hence it will not be taken into account in the evaluation. The most obvious change in the phase shift can be observed in the low and high frequency regions. At frequency 1 kHz, the phase shift gradually decreases over time. For this reason, it is not possible to use this system in its current state to detect pH changes.

**Figure 7:**
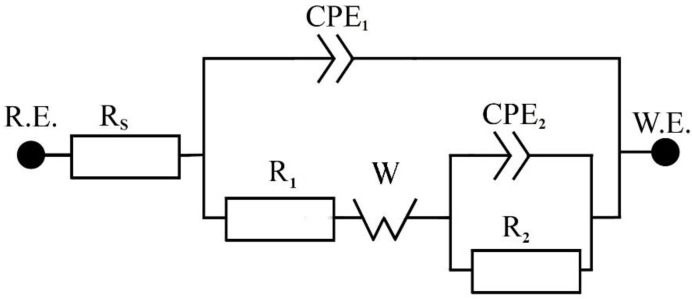
Schematic of EC used for modelling of EIS spectra for all samples.

For the evaluation of the impedance spectra, the equivalent circuit (EC) shown in Figure 7 was used for describing prepared systems. The following parts occur in the model:

- electrolyte resistance (*R_s_*), pore electrolyte resistance (*R*_1_), charge transfer resistance (*R*_2_);
- constant phase element (CPE_1_) with the coefficient (α_1_) corresponding to the capacitance of the nanotube wall;
- constant phase element (CPE_2_) with the coefficient (α_2_) corresponding to the capacitance of the inner interface;
- infinite Warburg impedance (*W*) for describing diffusion in pores with the Warburg coefficient (*σ*).

A CPE is used in EC as a substitute for capacitors, taking into account the non-ideal behavior of the system. This element is defined as Z = [C·(jω)^α^]^−1^, where α takes values in range 0 to 1. Values close to 0 correspond to the behavior of the resistor and values close to 1 to the capacitor [43,44]. The use of infinite Warburg impedance shows that the amount of Ru/RuO_2_ inside the nanotubes was sufficient to independently assume a role in the electrochemical processes. The Warburg coefficient was calculated using the Formula (3) [43]:

(3) $$\sigma =1/\left(\sqrt{2}\cdot W\right)$$

The first R-CPE term describes the passage of electrons through TiO_2_ nanotubes, followed by diffusion through the Ru/RuO_2_ layer, followed by electron diffusion through the electrochemical bilayer (the second R-CPE term).

The summary of the results of modelling the impedance spectra of all samples is in Table 5. As expected, electrolyte resistance (*R_s_*) values do not change significantly as a function of pH for any sample. There are also no significant changes in the CPE_1_ values, that characterize the capacitance of the wall of the nanotubes. This fact confirms that sufficiently similar TiO_2_ nanotubes have been prepared and that they are not significantly affected by changes in pH. *R*_1_ and *σ* elements present in the EC describe the behavior inside the nanotube. *R_1_* corresponds to the resistance of the electrolyte in the pore, infinite Warburg impedance (*W*) and corresponding coefficient *σ* show that the presence of Ru/RuO_2_ particles causes diffusion to be the main controlling process here. The values of these two elements are affected by the number of present particles. As their content increases, the pore becomes progressively clogged leading to an increase in resistance until complete blockage. Subsequently, a new charge transfer site is created. This process results in a significant shortening of the diffusion path. *W* reached the highest values in the sample PS 1200ox. This suggests that the high RuO_2_ content is conducive to a greater application of diffusion than in samples with unoxidized Ru (PS 600 and PS 1200). This trend is not confirmed by the sample with less RuO_2_ (PS 600ox), which is probably present in such a small amount that it does not allow the main diffusion process to take over. The presence of high amounts of RuO_2_ significantly affects the surface behavior also because this oxide is known for its supercapacitive behavior [36]. The values of charge transfer resistance (*R_2_*) do not vary significantly with pH or between the samples themselves. The magnitude of this element is mainly determined by the real reaction surface. The capacitance of the inner interface (CPE_2_) reaches the highest values in the PS 1200ox sample, which confirms that its properties are also influenced by the presence of RuO_2_.

**Figure 8:**
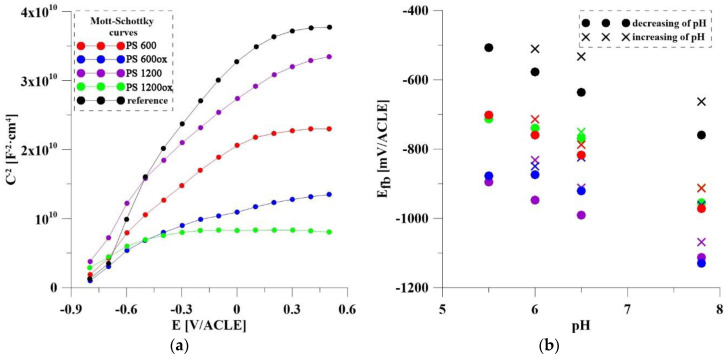
(**a**) Mott–Schottky curves of all samples; (**b**) dependence of *E*_fb_ on pH.

Another electrochemical method tested in this work for the detection of pH changes was the measurement of Mott–Schottky curves. Examples of measured MS at pH = 7.8 are shown in Figure 8a. Samples behaved like N-type semiconductors (positive trend), only PS 1200ox shows a change in the shape of the curves, which flatten out in the second half. This trend is due to the combined effects of the two semiconducting oxides. The value of the slopes did not vary significantly with pH, so it is not a suitable parameter for detecting pH changes. All the prepared systems showed a clear difference between the curves measured at pH = 7.8 and those measured at acidic pH (shift to the right, i.e., an increase of flat-band potential) which is the ideal behavior for the detection of inflammation. Considering the standard pH of human blood (7.35–7.45) and the precise regulation system, it can be said that already a drop to pH = 6.5 indicates that negative processes are occurring in the body. For this reason, this value will be taken as a signaling point of inflammation in the evaluation of the usefulness of the individual systems. This fact could be used to determine the unknown pH based on the flat-band potential (*E*_fb_), which can be calculated using Formula (1) [26] or evaluated graphically from the intersection of the slope with the zero-value of the squared capacitance. The graphically evaluated values of *E*_fb_ are shown in Figure 8b.

## 4. Conclusions

After comparing the results of individual samples within this work, it can be concluded that several promising systems for pH detection based on nanostructured alloy Ti-6Al-4V with deposited Ru/RuO_2_ particles have been proposed. The results showed that Mott–Schottky analysis is the most suitable method for pH changes detection.

As a potentiometric pH sensor, titania nanotubes with potentiostatic deposited Ru/RuO_2_ (−0.7 V/ACLE, 600 or 1200 s) should be used. However, for these systems, slightly higher stability has to be achieved. The method of measuring the impedance response gave inconsistent results. The phase shift was identified as a suitable pH dependent quantity. The charge transfer resistance was practically unchanged. In pH range 6.5–5.5, the phase shift varied for all samples with deposited Ru/RuO_2_. None of the samples gave sufficiently reproducible results, with subsequent pH increases from 5.5 to 7.8. Therefore, it is clear that in the current situation this method is not suitable for the used systems because of an extremely large amount of Cl^-^ in solution during measurement of pH = 5.5. In terms of possible conductometric pH sensors based on the EIS method, it would be best to steer development on the system with a high amount of oxidized Ru. Here the differences were noticeable at the highest frequencies (1 kHz), which are relatively fast to measure and would reduce the energy consumption of the pH sensor.

The Mott–Schottky analysis gave positive results for most systems. The decrease in pH was characterized by an increase of flat-band potential. The largest differences could always be observed between pH 7.8 and 6.5, which is the ideal situation for the detection of inflammation.

## Figures and Tables

**Table 1:** The measurement scheme of electrochemical response on change of pH.

Measurement	Conditions
***E*_OC_**	720 min
5 cycles	
***E*_OC_**	10 s
EIS	0 V/*E*_OC_, *E*_AC =_ 10 mV RMS, *f* = 60 kHz–2 mHz, 7 points to decade
***E*_OC_**	200 s
MS	−0.8 V/ACLE to 0.5 V/ACLE, step 0.1 V, *f* = 600 Hz, *E*_AC_ = 10 mV RMS

**Table 2:** Results of EDS analysis of prepared samples.

Content [wt.%]	Ti	Al	V	Ru	F
GS	5.0	0.2	0.3	94.3	0.2
CP	49.4	2.3	2.3	45.2	0.8
PS 600	84.0	5.4	4.1	2.5	4.0
PS 600ox	83.4	5.4	3.8	2.7	4.7
PS 1200	81.0	4.8	4.0	7.2	3.0
PS 1200ox	78.3	5.0	3.8	12.9	0.0

**Table 3:** The total calculated amount of deposited Ru by potentiostatic mode.

Sample/Weight of Ru	PS 600	PS 600ox	PS 1200	PS 1200ox
*m* [mg]	0.17	0.37	1.38	3.25

**Table 4:** Results of XPS analysis of representative PS 1200 and PS1200ox specimens.

Content [wt.%]	PS 1200	PS 1200ox
Ti	24.8	28.8
Al	8.0	10.3
Ru	67.2	60.9

**Table 5:** Results of modelling the impedance spectra for all samples.

	pH	*R*_s_ [Ω·cm^2^]	*R*_1_ [Ω·cm^2^]	CPE_1_ [S·s^α^/cm^2^]	α_1_	*W* [S·s^0.5^/cm^2^]	*R*_2_ [Ω·cm^2^]	CPE_2_ [S·s^α^/cm^2^]	α_2_	σ [Ω·s^1^·cm^2^]	χ^2^
**reference sample**	7.8	29.06	4.5 × 10^3^	1.1 × 10^−5^	0.91	4.8 × 10^−5^	1.5 × 10^5^	9.5 × 10^−5^	0.80	1.5 × 10^4^	8.5 × 10^−5^
	6.5	32.42	1.6 × 10^3^	1.1 × 10^−5^	0.91	3.4 × 10^−5^	5.5 × 10^5^	4.6 × 10^−5^	0.80	2.1 × 10^4^	2.4 × 10^−4^
	6.0	28.64	5.9 × 10^3^	1.7 × 10^−5^	0.90	4.1 × 10^−5^	9.2 × 10^4^	2.5 × 10^−5^	0.75	1.7 × 10^3^	4.7 × 10^−4^
	5.5	31.39	2.8 × 10^3^	1.1 × 10^−5^	0.92	5.2 × 10^−5^	1.9 × 10^5^	7.4 × 10^−5^	0.75	1.4 × 10^4^	1.8 × 10^−4^
	6.0	31.41	3.2 × 10^3^	1.2 × 10^−5^	0.91	6.4 × 10^−5^	1.1 × 10^5^	9.9 × 10^−5^	0.75	1.1 × 10^4^	1.2 × 10^−4^
	6.5	30.45	3.5 × 10^3^	1.2 × 10^−5^	0.91	6.6 × 10^−5^	9.9 × 10^4^	1.0 × 10^−4^	0.75	1.1 × 10^4^	1.4 × 10^−4^
	7.8	29.06	4.5 × 10^3^	1.1 × 10^−5^	0.91	4.8 × 10^−5^	1.5 × 10^5^	9.5 × 10^−5^	0.80	1.5 × 10^4^	8.5 × 10^−5^
**PS 600**	7.8	30.99	0.0	4.9 × 10^−6^	1.00	2.8 × 10^−4^	2.6 × 10^5^	2.3 × 10^−5^	0.75	2.5 × 10^3^	1.9 × 10^−4^
	6.5	29.14	4.0 × 10^3^	1.6 × 10^−5^	0.90	3.5 × 10^−4^	1.1 × 10^5^	2.1 × 10^−5^	0.75	2.0 × 10^3^	3.3 × 10^−4^
	6.0	28.64	5.9 × 10^3^	1.7 × 10^−5^	0.90	4.1 × 10^−4^	9.2 × 10^4^	2.5 × 10^−5^	0.75	1.7 × 10^3^	4.7 × 10^−4^
	5.5	28.26	7.9 × 10^3^	1.8 × 10^−5^	0.90	4.0 × 10^−4^	8.5 × 10^4^	2.9 × 10^−5^	0.75	1.8 × 10^3^	7.4 × 10^−4^
	6.0	27.90	7.5 × 10^3^	1.7 × 10^−5^	0.90	4.0 × 10^−4^	8.2 × 10^4^	3.5 × 10^−5^	0.75	1.8 × 10^3^	6.3 × 10^−4^
	6.5	27.36	7.0 × 10^3^	1.6 × 10^−5^	0.91	4.2 × 10^−4^	7.8 × 10^4^	3.8 × 10^−5^	0.75	1.7 × 10^3^	4.6 × 10^−4^
	7.8	27.03	1.0 × 10^4^	1.6 × 10^−5^	0.91	4.0 × 10^−4^	8.2 × 10^4^	3.3 × 10^−5^	0.75	1.8 × 10^3^	5.5 × 10^−4^
**PS 600ox**	7.8	31.93	6.6 × 10^4^	1.8 × 10^−5^	0.91	4.3 × 10^−5^	2.1 × 10^5^	7.8 × 10^−6^	0.80	1.7 × 10^4^	1.2 × 10^−4^
	6.5	30.60	4.6 × 10^4^	2.0 × 10^−5^	0.90	4.8 × 10^−5^	1.7 × 10^5^	8.1 × 10^−6^	0.80	1.5 × 10^4^	1.1 × 10^−4^
	6.0	29.38	3.9 × 10^4^	2.1 × 10^−5^	0.89	4.7 × 10^−5^	1.6 × 10^5^	7.6 × 10^−6^	0.80	1.5 × 10^4^	9.4 × 10^−5^
	5.5	29.80	3.0 × 10^4^	2.2 × 10^−5^	0.89	4.6 × 10^−5^	1.7 × 10^5^	7.0 × 10^−6^	0.80	1.5 × 10^4^	9.4 × 10^−5^
	6.0	28.70	2.5 × 10^4^	2.1 × 10^−5^	0.89	4.3 × 10^−5^	1.5 × 10^5^	8.7 × 10^−6^	0.80	1.6 × 10^4^	9.7 × 10^−5^
	6.5	28.01	2.2 × 10^4^	2.0 × 10^−5^	0.90	4.1 × 10^−5^	1.3 × 10^5^	1.0 × 10^−5^	0.80	1.7 × 10^4^	1.0 × 10^−4^
	7.8	27.33	2.8 × 10^4^	1.7 × 10^−5^	0.91	3.9 × 10^−5^	1.2 × 10^5^	1.0 × 10^−5^	0.80	1.8 × 10^4^	1.0 × 10^−4^
**PS 1200**	7.8	33.41	3.5 × 10^3^	1.7 × 10^−5^	0.86	6.2 × 10^−5^	5.0 × 10^5^	7.9 × 10^−5^	0.81	1.1 × 10^4^	2.6 × 10^−4^
	6.5	30.76	2.3 × 10^3^	1.6 × 10^−5^	0.88	7.4 × 10^−5^	1.9 × 10^5^	1.7 × 10^−4^	0.90	9.6 × 10^3^	2.2 × 10^−4^
	6.0	29.77	2.0 × 10^3^	1.5 × 10^−5^	0.88	7.6 × 10^−5^	1.3 × 10^5^	2.0 × 10^−4^	0.91	9.3 × 10^3^	2.2 × 10^−4^
	5.5	29.68	1.9 × 10^3^	1.5 × 10^−5^	0.88	8.1 × 10^−5^	1.3 × 10^5^	1.9 × 10^−4^	0.91	8.8 × 10^3^	2.3 × 10^−4^
	6.0	28.79	2.2 × 10^3^	1.5 × 10^−5^	0.89	7.5 × 10^−5^	1.3 × 10^5^	1.9 × 10^−4^	0.89	9.5 × 10^3^	1.8 × 10^−4^
	6.5	27.67	2.7 × 10^3^	1.4 × 10^−5^	0.89	7.2 × 10^−5^	1.3 × 10^5^	1.8 × 10^−4^	0.87	9.9 × 10^3^	1.6 × 10^−4^
	7.8	27.32	5.2 × 10^3^	1.4 × 10^−5^	0.89	5.7 × 10^−5^	1.3 × 10^5^	1.2 × 10^−4^	0.80	1.2 × 10^4^	2.3 × 10^−4^
**PS 1200ox**	7.8	33.00	57.7	1.3 × 10^−5^	0.88	6.7 × 10^−3^	8.6 × 10^5^	9.6 × 10^−4^	0.84	1.1 × 10^2^	8.6 × 10^−5^
	6.5	28.30	90.6	1.5 × 10^−5^	0.87	9.3 × 10^−3^	1.1 × 10^5^	2.6 × 10^−3^	0.93	7.6 × 10^1^	8.6 × 10^−5^
	6.0	31.00	65.7	1.4 × 10^−5^	0.88	9.8 × 10^−3^	1.7 × 10^5^	3.0 × 10^−3^	0.92	7.2 × 10^1^	9.6 × 10^−5^
	5.5	29.30	75.9	1.4 × 10^−5^	0.87	1.1 × 10^−3^	1.6 × 10^5^	2.1 × 10^−3^	0.88	6.4 × 10^2^	1.8 × 10^−4^
	6.0	29.00	85.6	1.4 × 10^−5^	0.87	8.4 × 10^−3^	1.3 × 10^5^	2.5 × 10^−3^	0.92	8.4 × 10^1^	1.4 × 10^−4^
	6.5	28.30	90.6	1.5 × 10^−5^	0.97	9.3 × 10^−3^	1.1 × 10^5^	2.6 × 10^−3^	0.93	7.6 × 10^1^	8.6 × 10^−5^
	7.8	26.90	96.9	1.3 × 10^−5^	0.88	8.0 × 10^−3^	1.7 × 10^5^	2.5 × 10^−3^	0.93	8.8 × 10^1^	9.7 × 10^−5^

## Data Availability

Data is contained within the article.
